# Phylogenetic diversity and *in situ* detection of eukaryotes in anaerobic sludge digesters

**DOI:** 10.1371/journal.pone.0172888

**Published:** 2017-03-06

**Authors:** Miri Matsubayashi, Yusuke Shimada, Yu-You Li, Hideki Harada, Kengo Kubota

**Affiliations:** 1 Department of Civil and Environmental Engineering, Tohoku University, Sendai, Miyagi, Japan; 2 Department of Frontier Science for Advanced Environment, Tohoku University, Sendai, Miyagi, Japan; 3 New Industry Creation Hatchery Center, Tohoku University, Sendai, Miyagi, Japan; Austrian Institute of Technology, AUSTRIA

## Abstract

Eukaryotic communities in aerobic wastewater treatment processes are well characterized, but little is known about them in anaerobic processes. In this study, abundance, diversity and morphology of eukaryotes in anaerobic sludge digesters were investigated by quantitative real-time PCR (qPCR), 18S rRNA gene clone library construction and catalyzed reporter deposition-fluorescence in situ hybridization (CARD-FISH). Samples were taken from four different anaerobic sludge digesters in Japan. Results of qPCR of rRNA genes revealed that *Eukarya* accounted from 0.1% to 1.4% of the total number of microbial rRNA gene copy numbers. The phylogenetic affiliations of a total of 251 clones were Fungi, Alveolata, Viridiplantae, Amoebozoa, Rhizaria, Stramenopiles and Metazoa. Eighty-five percent of the clones showed less than 97.0% sequence identity to described eukaryotes, indicating most of the eukaryotes in anaerobic sludge digesters are largely unknown. Clones belonging to the uncultured lineage LKM11 in Cryptomycota of Fungi were most abundant in anaerobic sludge, which accounted for 50% of the total clones. The most dominant OTU in each library belonged to either the LKM11 lineage or the uncultured lineage A31 in Alveolata. Principal coordinate analysis indicated that the eukaryotic and prokaryotic community structures were related. The detection of anaerobic eukaryotes, including the members of the LKM11 and A31 lineages in anaerobic sludge digesters, by CARD-FISH revealed their sizes in the range of 2–8 μm. The diverse and uncultured eukaryotes in the LKM11 and the A31 lineages are common and ecologically relevant members in anaerobic sludge digester.

## Introduction

Anaerobic sludge digestion is a well-established waste treatment process. The benefits of the anaerobic sludge digestion are energy production mostly in the form of methane, sludge volume reduction and sludge stabilization. Anaerobic digestion is a complex and multistep process in which various microorganisms are involved in the degradation of organic matter. One of the well-known microbial interactions in the anaerobic environment is the syntrophic association between fatty-acid-degraders and hydrogenotrophic methanogens. As a result, many researchers have focused their investigation on the prokaryotic community structure in anaerobic sludge digesters [1,2]. On the other hand, less attention has been paid to study eukaryotes in anaerobic sludge digesters. The role of eukaryotes has already been highlighted in the important areas of aerobic treatment processes, such as process performance, predation on bacteria and excess sludge production [3]. Therefore, it is also important to investigate the contribution of eukaryotes in anaerobic digestion by studying their diversity, population, roles and functions.

Microscopic observation has always been a popular way for detection, identification, and enumeration of eukaryotes due to their large size. However, microscopy is not always a preferable way to study eukaryotes because of their lower detectability. For example, *Minidiscus* and *Eucampia* cells are often overlooked by microscopic observations [4]. Alternatively, molecular approaches such as rRNA gene based identification have been recently used to understand eukaryotic diversity in natural and engineered environments [5–7].

Here, we investigated the phylogenetic diversity and abundance of eukaryotes in anaerobic sludge digesters. Furthermore, eukaryotes were morphologically identified by catalyzed reporter deposition—fluorescence in situ hybridization (CARD-FISH). The results provide a framework for future ecological and functional studies of eukaryotes in anaerobic sludge digesters.

## Materials and methods

### Sample collection and DNA extraction

Sludge samples were taken from mesophilic anaerobic digesters at four different sewage treatment plants (indicated as N, K, S and M plants) in Japan. Samplings were permitted by the authorities concerned about sewage treatment in Nagaoka city for the Nagaoka chuo sewage treatment plant (the N plant), Takamatsu city for a sewage treatment plant (the K plant), Miyagi prefecture for a sewage treatment plant (the S plant), and Morioka city for the Nakagawara sewage treatment plant (the M plant). The N and K treatment plants employ conventional activated sludge processes whereas the S sewage works operates a pseudo anaerobic-oxic process and an anaerobic-anoxic-oxic process for sewage treatment. The M sewage treatment plant was equipped with a trickling filter process. All digesters are fed with the mixture of primary sludge from primary sedimentation tanks and waste sludge from sewage treatment processes. Further information on anaerobic sludge digesters are shown in S1 Table. Sludge samples from the S plant were collected twice at different time intervals (2013 & 2014; named as S13 & S14). One sludge sample was collected from the rest of the other digesters. All five sludge samples were washed with phosphate-buffered saline (137 mM NaCl, 8.10 mM Na_2_HPO_4_, 2.68 mM KCl, 1.47 mM KH_2_PO_4_ [pH7.4]) immediately after sampling, and stored at -20°C for DNA extraction. DNA was extracted using ISOIL for Beads Beating Kit (NIPPON GENE, Tokyo, Japan).

### Quantitative real-time PCR

Copy numbers of each domain, i.e., *Bacteria*, *Archaea* and *Eukarya*, was determined by quantitative real-time PCR using SYBR *Premix Ex Taq* (TaKaRa, Tokyo, Japan) with a LightCycler instrument (Roche, Basel, Switzerland). Primer sets used for *Bacteria*, *Archaea* and *Eukarya* were Eub338f(ver.1-ver.4)/907r, Arc109f/Arc912r, Euk299f/Euk526r, respectively (S2 Table). Purity of the PCR products was verified by melting analysis and agarose gel electrophoresis. All tests were performed in triplicate. The ratio of the number of rRNA gene copies of each microbial domain was calculated considering the sum of the rRNA gene copies from all domains as total abundance. Note that the number of rRNA operons per cells varies among eukaryotes [8] as well as prokaryotes [9], and therefore the ratios are based on gene copy numbers, not their cell numbers.

### Eukaryotic clone library construction

Near-full-length eukaryotic 18S rRNA genes were amplified using *TaKaRa Ex Taq* Hot Start Version (TaKaRa) with the EukA/EukB eukaryotic universal primer set (S2 Table). PCR condition was as follows: initial denature at 98°C for 2 min, followed by 25 cycles of 98°C for 10 sec, 55°C for 30 sec, and 72°C for 150 sec, and final extension at 72°C for 10 min. After purification with MicroSpin S-400 HR Columns (GE Healthcare, Little Chalfont, U.K) and MinElute PCR Purification Kit (Qiagen, Tokyo, Japan), the PCR products were cloned using TOPO 2.1 TA Cloning Kit (Invitrogen, Carlsbad, USA). Approximately 50 clones from each sample (264 clones in total) were picked and the near-full-length sequences of 18S rRNA genes of all clones were determined. The clones were classified into operational taxonomic unit (OTU) with a threshold of 98% sequence identity. The sequences were analyzed using the ARB software package with the SILVA database [10] and BLAST search in the National Center for Biotechnology Information database. Principal coordinate analysis (PCoA) was conducted using Mothur software [11]. The sequences were deposited in the DDBJ/NCBI/EMBL database under Accession Numbers LC108996 –LC109102 (S3 Table).

### Pyrosequencing of prokaryotic 16S rRNA gene

The V3-V4 region of prokaryotic 16S rRNA gene was amplified using AmpliTaq Gold DNA polymerase, LD (Low DNA) with the 341f-806rmix primer set (S2 Table). The primers were modified to improve the coverage. PCR condition was as follows: initial denature at 95°C for 10 min, followed by 25 cycles of 95°C for 30 sec, 50°C for 30 sec, and 72°C for 45 sec. After purification with MicroSpin S-400 HR Columns (GE Healthcare, Little Chalfont, U.K) and MinElute PCR Purification Kit (Qiagen, Tokyo, Japan), the PCR products were subjected to pyrosequencing using emPCR kit (Lib-L) and GS Jr. instrument (Roche) according to the manufacturer’s instruction. The obtained sequences were analyzed using QIIME software [12] with Greengenes 13_8 dataset.

### Fluorescence In Situ Hybridization (FISH)

A portion of each sludge sample was fixed with 2.4% paraformaldehyde, and stored in ethanol/PBS solution at -20°C. The FISH analysis was performed as described previously [13] with EUK516 labeled with Cy5 (S2 Table). Formamide concentration was adjusted to 5%.

### Probe design and validation

Oligonucleotide probes targeting the members of interests were designed using ARB software [14]. The designed probes were first evaluated using mathFISH [15]. If a thermodynamic value of a designed probe did not reach to the threshold (ΔG°_overall_ = -13 kJ/mol), the probe was elongated or locked nucleic acid (LNA) was introduced [13]. The designed probes were eventually validated as described elsewhere [16]. The designed probe sequences are shown in S2 Table.

### Catalyzed Reporter Deposition-FISH (CARD-FISH)

CARD-FISH was conducted as described previously [17]. Briefly, the paraformaldehyde fixed samples were subjected to a 10-well glass slide and air-dried. After dehydration with ethanol series (50, 80, and 99.5% ethanol for 5, 1, and 1 min, respectively), the samples were treated with chitinase (1 mg/mL in PBS with 1% sodium dodecyl sulfate [SDS]) for 10 min at 30°C for permeabilization [18], followed by washing in TNT (100 mM Tris-HCl [pH7.5], 150 mM NaCl, 0.05% Tween-20) buffer (15 min, room temperature [RT: kept at 20–25°C by air-conditioner]) and ultra-pure water (1 min, RT), dehydrated in 95% ethanol (1min, RT), and air-dried. Endogenous peroxidases were inactivated by treating the samples with 0.5% hydrogen peroxide in methanol (30 min, RT) [19]. Hybridization was carried out in buffer (20 mM Tris-HCl [pH7.5], 0.9 M NaCl, 30–40% formamide, 1% blocking reagent, 10% dextran sulfate, 0.01% SDS) containing 0.1 μM of the horseradish peroxidase (HRP)-labeled probe for 3–6 hours at 40°C. Subsequently, excess probes were removed by immersing the glass slides in buffer (20 mM Tris-HCl [pH7.5], 0.9 M NaCl, 30–40% formamide, 5 mM EDTA, 0.01% SDS) for 20 minutes at 42°C. For tyramide signal amplification, the glass slides were first immersed into TNT buffer for 15 min at RT. After removing buffer around each well, the samples were incubated with TSA working solution (1 volume of tyramide-FITC or tyramide-Cy5, 37.5 volumes of amplification diluent, 12.5 volumes of 40% dextran sulfate, 0.5 volumes of 10% blocking reagent) for 15 min at 37°C. Then, the slides were immersed in TNT buffer (15 min, RT), ultra-pure water (1 min, RT), and ethanol (1 min, RT), and subsequently air-dried. For dual staining, CARD-FISH with a group specific probe was initially carried out. After quenching HRP activity of the hybridized probes with hydrogen peroxide, the second round of CARD-FISH with the EUK516 probe was conducted. The samples were embedded in ProLong Gold Antifade Reagent with DAPI (Invitrogen), and subjected to microscopic observation. For microscopy, Axio Imager.M2 epifluorescent microscope equipped with AxioCam HRM (Carl Zeiss) was used.

## Results

### Phylogenetic analysis of eukaryotes

A total of 264 clones were sequenced, in which 13 clones belonged to the domain *Archaea*. Eventually, five eukaryotic clone libraries were constructed from 251 clones, consisting of 45 to 55 clones in each library. The diversity indices and eukaryotic composition of each library are shown in Table 1 and S3 Table. Fourteen to twenty-eight OTUs were retrieved with a 98% sequence identity threshold in each library. The Chao1 nonparametric estimator indicated that there should be 34–77 OTUs in the samples.

**Table 1:** Eukaryotic community compositions and diversity indices based on 18S rRNA gene clone libraries.

Sample name	S13	S14	N	K	M
No.of sequences	52	48	55	51	45
No. of OTU	28	22	16	14	27
Chao1	61	34	77	46	54
Coverage	0.46	0.54	0.71	0.73	0.40
Evenness	0.88	0.90	0.74	0.79	0.93
**Fungi**					
Cryptomycota (LKM11)	34.6^a^	41.7	87.3	62.7	20.0
Ascomycota	17.3	20.8	5.5	2.0	13.3
Basidiomycota	1.9		1.8		6.7
Zoopagales	3.8				
Chytridomycota		4.2			
LKM15			1.8		
**Alveolata**					
A31	21.2	18.8		21.6	
Other	1.9	4.2			4.4
**Viridiplantae**	9.6	4.2	1.8	11.8	28.9
**Rhizaria**		2.1			2.2
**Stramenopiles**	1.9				
**Amoebozoa**					6.7
**Metazoa**	7.7	4.2	1.8	2.0	17.8

^a^ Frequencies (%) in each library

**Fig 1:**
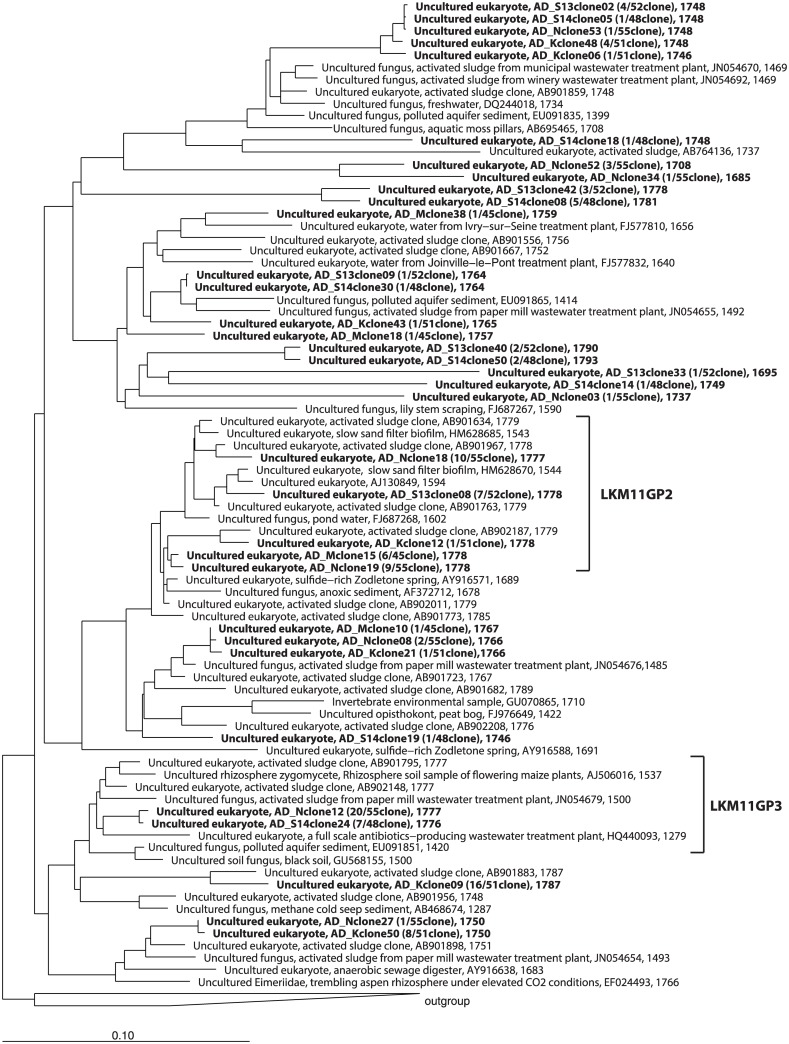
Phylogenetic tree of the LKM11 lineage in Fungi based on a comparative analysis of 18S rRNA gene sequences. The OTUs obtained in this study are shown in bold type in the tree. The tree also indicates the coverage of oligonucleotide probes, LKM11GP2 and LKM11GP3, designed in this study.

**Fig 2:**
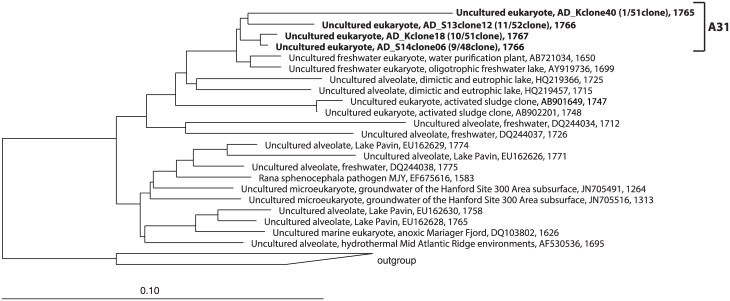
Phylogenetic tree of the A31 lineage in Alveolata based on a comparative analysis of 18S rRNA gene sequences. The OTUs obtained in this study are shown in bold type in the tree. The tree also indicates the coverage of the A31 probes designed in this study.

The identified clones belonged to Fungi (66%), Alveolata (14%), Viridiplantae (11%), Metazoa (6%), Amoebozoa (1%), Rhizaria (1%), and Stramenopiles (1%) (S1 Fig). Cryptomycota and Ascomycota (in Fungi), Viridiplantae, and Metazoa were found in all samples. Clones belonging to the uncultured fungal lineage LKM11 in Cryptomycota were dominant members in all libraries (N library: 9 OTUs, 48/55 clones, K library: 7 OTUs, 32/51 clones, S13 library: 6 OTUs, 18/52 clones, S14 library: 9 OTUs, 20/48 clones) except for the clone library of the M plant, in which Chlorophyta in Viridiplantae (9 OTUs, 13/45 clones) was the most predominant (S3 Table). The most predominant OTU in the N, K, and M libraries belonged to the LKM11 lineage (20 clones, 16 clones, and 6 clones, respectively). The most dominant OTU in the S13 and S14 libraries belonged to the A31 lineage in Alveolata (11 clones and 9 clones, respectively). An OTU belonging to the A31 lineage was the second most dominant OTU in the K library (10 clones). The phylogenetic trees of the LKM11 and A31 lineages are shown in Figs 1 and 2, respectively. OTUs belonging to family Dipodascaceae in Ascomycota (in Fungi) and family Chlorellaceae in Chlorophyta (in Viridiplantae) were also found among the libraries. OTUs belonging to Zoopagales, Chytridomycota and the LKM15 lineage in Fungi, Stramenopiles and Amoebozoa were specifically found in one of the libraries. Anaerobic fungi belonging to Neocallimastigomycota [20] was not found in the libraries.

### Prokaryotic community structure

After denoising and chimera checking, a total number of 45,360 sequences were used for describing prokaryotic community structure. The numbers of sequence reads, diversity indicies, and prokaryotic community compositions are shown in S4 Table.

### Abundance of eukaryotic, bacterial, and archaeal rRNA genes

Quantitative real-time PCR was conducted to quantify the number of rRNA gene copies of each domain in the sludge samples. Copy numbers of the small-subunit of the rRNA gene are shown in Table 2. Bacterial rRNA gene copies were the most abundant in all samples, ranging from 90.6% to 97.3% of the total rRNA gene copy number. The relative abundances of archaeal rRNA genes were from 2.0% to 9.1%, and eukaryotic rRNA gene copy numbers accounted for 0.1%–1.4% of the total rRNA gene copy number. It should be noted that the number of rRNA gene copies is heterogeneous among eukaryotes [8] as well as prokaryotes [9]. It has been reported that rRNA gene copy number has correlations with genome size (plants and animals) [8] and cell length (phytoplankton) [21] in eukaryotes. The numbers of rRNA gene copy numbers of eukaryotes retrieved in this study are unknown. It should be in mind that the ratio calculated here is not a population of each domain at cellular level.

**Table 2:** Copy numbers and proportions of rRNA genes of each domain as determined by quantitative PCR.

Sample name	Unit	*Eukarya*	*Bacteria*	*Archaea*
S13	copies/ng-DNA	1.4×10^3^±3.0×10^2^	2.7×10^5^±4.2×10^4^	7.0×10^3^±4.4×10^3^
	%	**0.5**	**97.4**	**2.5**
S14	copies/ng-DNA	7.8×10^2^±1.3×10^2^	8.3×10^5^±3.7×10^5^	2.3×10^4^±1.3×10^4^
	%	**0.1**	**97.3**	**2.7**
N	copies/ng-DNA	1.8×10^3^±1.9×10^2^	1.3×10^5^±6.2×10^4^	2.6×10^3^±9.8×10^2^
	%	**1.4**	**97.9**	**2.0**
K	copies/ng-DNA	8.2×10^2^±3.6×10^1^	9.4×10^4^±1.0×10^4^	2.0×10^3^±8.6×10^2^
	%	**0.9**	**97.9**	**2.0**
M	copies/ng-DNA	3.1×10^2^±1.3×10^2^	4.6×10^4^±7.2×10^3^	4.6×10^3^±2.1×10^3^
	%	**0.6**	**90.6**	**9.1**

### In situ detection of eukaryotes

**Fig 3:**
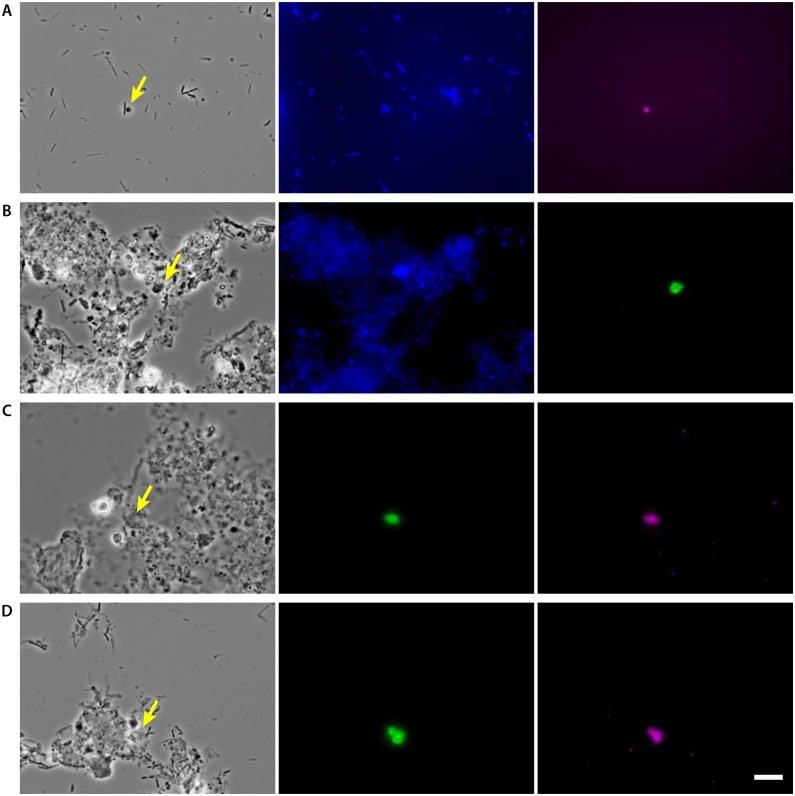
Detection of eukaryotes in anaerobic sludge digester by CARD-FISH. A & B: Probe-derived signal after hybridization with the HRP-labeled EUK516 probe and tyramide signal amplification with Cy5-labeled tyramide (A) and FITC-labeled tyramide (B). Photomicrographs of phase contrast (left), DAPI (middle) and EUK516-derived signals (right) showing identical fields. C & D: Dual staining of a group specific probe (FITC-labeled tyramide) and the EUK516 probe (Cy5-labeled tyramide). Photomicrographs of phase contrast (left), a group specific probe, LKM11GP2 (C) and A31 (D) (middle) and EUK516-derived signals (right) showing identical fields. Bar represents 10 μm.

Initially, FISH with the Cy5-labeled EUK516 probe, designed to detect most of *Eukarya*, was conducted; nevertheless, reliable fluorescent signals were not observed most likely due to low rRNA contents of the cells [22]. Recently developed in situ DNA-HCR was also performed to enhance the signal detection [23]. However, reliable signals were still not obtained. Eventually, CARD-FISH was conducted after the endogenous peroxidase inactivation with hydrogen peroxide in methanol, but a very small number of cells were detected. The number of detectable cells increased after the cell wall treatment with chitinase (1 mg/ml in PBS with 1% SDS) [18]. The signals were strong and reliable and were obtained from different cell sizes ranging from 2 to 8 μm (Fig 3).

The detection of major eukaryotes in our clone libraries was conducted with group specific probes designed in this study. Two probes for the LKM11 lineage and one probe for the A31 lineage were designed to cover the major clones retrieved in this study (Figs 1 and 2 and S2 Table). The detection of the members of the LKM11 lineage as well as those of the A31 lineage was successful, and these signals were confirmed by dual staining with the EUK516 probe.

## Discussion

In this study, we investigated eukaryotes in anaerobic sludge digesters by using 18S rRNA gene sequence information to understand their diversity, abundance and morphology. Very little is known about the anaerobic eukaryotic community as compared to those in aerobic processes. To our knowledge, there is only one report on clone library of an anaerobic sludge digester [24], but the study mainly investigated a microeukaryotic community in anaerobic sulfide and sulfur-rich springs. Therefore, this is the first systematic study to investigate eukaryotic communities in anaerobic sludge digester by comparative analysis based on 18S rRNA gene sequence information. For this purpose, we constructed the clone libraries by sequencing near-full-length 18S rRNA gene sequences to increase the number of reliable environmental eukaryotic sequences in the database. High-throughput sequencing with next-generation sequencers is a common and faster way for microbial community analysis nowadays, but a substantial database with reliable sequences is essential. The sequences obtained in this study are useful for database construction and high-throughput eukaryotic community analysis of anaerobic sludge samples.

Out of a total of 251 clones, only 39 clones (15.5%) showed ≥97.0% sequence identity to described eukaryotes (S1 Fig). All clones classified as metazoan showed high sequence identity (≥97.0%) to existing eukaryotes database. The remaining 212 clones (84.5%) had <97.0% sequence identity to described eukaryotes, indicating the most of eukaryotes in the anaerobic sludge samples investigated in this study are physiologically and metabolically unknown. The majority of these undescribed eukaryotes belonged to Fungi (151/212 clones, 71.7%), in which 127 clones belonged to the uncultured LKM11 lineage belonging to the fungi group Cryptomycota. The second dominant group was Alveolata (34/212 clones, 16.0%), and 31 clones belonged to the uncultured A31 lineage.

The anaerobic sludge digesters, from which we took samples, treat a mixture of primary and waste sludges. The preexisting eukaryotic community in the feed sludge could have some impact on the microbial community and performance of the anaerobic digesters. Matsunaga et al. [6] investigated the eukaryotic community in activated sludge processes and created 18S rRNA gene clone libraries. After analyzing a total of 834 clones, Alveolata (41%), Fungi (33%), Rizaria (11%) and metazoan (11%) were found to be major communities in the aerobic processes. The metazoan (Nematoda, Gastrotricha, Rotifera) and the protozoan (those in subclass Peritrichia and Cercozoa in Rhizaria) are dominant in activated sludge. However, their numbers in the anaerobic sludge samples were very insignificant, which indicates their inability to thrive in the anaerobic environment.

The dominant eukaryotic clones in the anaerobic sludge that belonged to the LKM11 lineage were also major fungi found in activated sludge (approximately 25% of the total clones). It can be thought that debris from activated sludge resulted in the predominance of the clones in this lineage. Nevertheless, it is improbable to think that only the members of the LKM11 lineage, unlike other predominant members in the activated sludge samples, somehow only retain their cells and DNAs during anaerobic digestion. Rather, it is more likely that the LKM11 lineage could have active participation in the anaerobic digestion process. As mentioned earlier, Luo et al. [24] also confirmed the presence of LKM11 lineage in an anaerobic sludge digester and Dawson and Pace [25] reported their presence in anoxic sediments. Additionally, Brad et al. [26] detected LMK11 lineage in anaerobic groundwater polluted with landfill leachate, which indicates their survival and propagation in anaerobic environment.

In this study, the diversity and variety of eukaryotes belonging to this lineage was retrieved from anaerobic sludge digester (Fig 1). Some of the clone sequences had high sequence identity to the sequences retrieved from activated sludge processes, suggesting that the genetic diversity of this lineage in anaerobic sludge digester is most likely affected by those in their feed sludge. Functions and roles of eukaryotes in the LKM11 lineage in both aerobic and anaerobic ecosystems are still largely unknown. Some members of the LKM11 lineage in freshwater are expected to be related to the decomposition of detritus or phytoplanktonic organisms [27]. The large number of diverse clones retrieved in this study indicated that the members of LKM11 are common facultative anaerobic eukaryotes and may have a variety of important functions in anaerobic sludge digestion processes. Further studies are necessary to elucidate this expectation; nevertheless, methods such as isotope probing are difficult to apply because we have no idea about their physiologies at this moment.

The uncultured A31 lineage is in Perkinsidae of Alveolata, and their functions are also still unknown. The OTUs belonging to this lineage were dominant in the S13 and S14 samples and the second abundant OTU in the K sample (Fig 2). Phylogenetic distances of major OTUs (S13clone12, S14clone06 and Kclone18) are more than 97%, indicating the diversity of eukaryotes in this lineage in anaerobic sludge digester is less. However, none of the clones belonging to this lineage were detected in the N and M samples. Thus the eukaryotes belonging to A31 lineage may require certain anaerobic conditions for survival and growth. Further investigation is required in this aspect.

Very limited information on anaerobic or facultative anaerobic eukaryotic clones is available to date. Therefore, we attempted to grow and isolate some of the important eukaryotic species, and obtained a *Candida* strain from an anaerobic digester sample (data not shown). *Candida* sp. in Ascomycota of Fungi, also found in the clone libraries, can grow on PG broth and YNBG broth in an anaerobic condition although the growth yields were lower than those in aerobic conditions [28]. Therefore, they contribute to the degradation of some organic matter in anaerobic environments. The two enzymes (aspartyl proteinases and phospholipases) secreted by *C*. *albicans* are known to contribute to pathogenicity [29]. Thus, it is speculated that the member of *Candida* could also be contributing to hydrolysis of organic matter in anaerobic sludge digestion processes.

Overall community structures of both eukaryotes and prokaryotes were compared by PCoA (S3 Fig). The S13, S14 and K samples are closely related, and the N sample and the M sample are individually standing. It is expected that the community structures of the S13 and S14 samples are similar because the samples were taken from the same reactor. On the other hand, the location of the K sewage works is very far from that of the S sewage works (more than 1,000 km away), and these two sewage works employ different activated sludge processes. Therefore, their closeness in PCoA plots was unexpected. This result suggests that prokaryotic and eukaryotic community structures are not individually composed; rather, the both communities are closely related and may be complementing to establish a single microbial community structure for anaerobic digestion.

Our CARD-FISH experiments revealed that the eukaryotes were not abundantly present in the sludge samples. Smaller size (2–8 μm) and less morphological differentiation of eukaryotic cells in anaerobic sludge digester made it difficult to distinguish them in the sludge samples. The detection of the members in the LKM11 and A31 lineages indicated that the morphologies were different from those found in lake [18], and the sizes were approximately 5–8 μm. Because the functions of eukaryotes belonging to the LKM11 and A31 lineages are largely unknown, the successful detection of these eukaryotes can lead to future functional analyses of these eukaryotes using single-cell microbiology tools (e.g., microautoradiography-FISH, FISH with NanoSIMS, single-cell genomics after fluorescently activated cell sorting, etc.) [30–32].

## Conclusions

This study revealed the diversity of eukaryotes in anaerobic sludge digesters by constructing 18S rRNA gene clone library. In addition, the eukaryotic cells were morphologically identified by CARD-FISH. The results of this study also concluded that the most of eukaryotes identified are physiologically not described previously. The diverse and uncultured eukaryotes in the LKM11 and the A31 lineages are found to be common and most likely ecologically relevant members in anaerobic sludge digester. In addition, the relationship between the eukaryotic and prokaryotic community structures was suggested. The majority of eukaryotes are still uncultured and their functions are largely unknown; and therefore further investigation on the metabolic functions of these eukaryotes, especially those in the LKM11 and the A31 lineages, are necessary to understand their contributions to anaerobic sludge digestion processes.

## Supporting information

S1 Fig Kingdom/Superphylum level eukaryotic community composition. With all clone sequences (a) and community compositions based on clones with ≥97% (b) or <97% (c) sequence identity to described eukaryotes.
(PDF)

S2 Fig Prokaryotic community compositions of five sludge samples. (PDF)

S3 Fig Principal Coordinate Analysis (PCoA) of eukaryotic (a) and prokaryotic (b) community structures. (PDF)

S1 Table Parameters of full-scale anaerobic digesters. (PDF)

S2 Table Primers and probes used in this study. (PDF)

S3 Table List of Operational Taxonomic Units (OTUs) (PDF)

S4 Table The numbers of sequence reads and diversity indices of prokaryotic community. (PDF)
