# Supplementary material for: Phylogenetic diversity and in situ detection of eukaryotes in anaerobic sludge digesters
Source: PLoS One. 2017 Mar 6;12(3):e0172888. doi: 10.1371/journal.pone.0172888 (PMC5338771; doi:10.1371/journal.pone.0172888)
Supplement: S1 Fig — With all clone sequences (a) and community compositions based on clones with ≥97% (b) or <97% (c) sequence identity to described eukaryotes. (PDF) [file pone.0172888.s001.pdf]

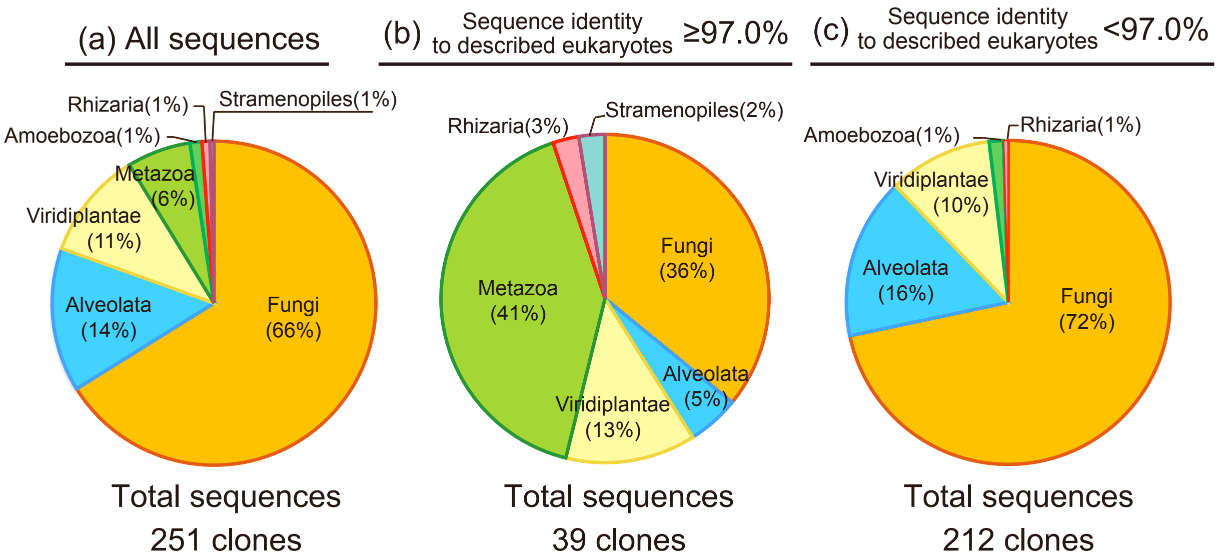

S1 Fig. Kingdom/superphylum level eukaryotic community composition. With all clone sequences (a) and community compositions based on clones with ≥97% (b) or <97% (c) sequence identity to described eukaryotes.
