# Supplementary figures and images for: Phylogenetic diversity and in situ detection of eukaryotes in anaerobic sludge digesters
Source: PLoS One. 2017 Mar 6;12(3):e0172888. doi: 10.1371/journal.pone.0172888 (PMC5338771; doi:10.1371/journal.pone.0172888)

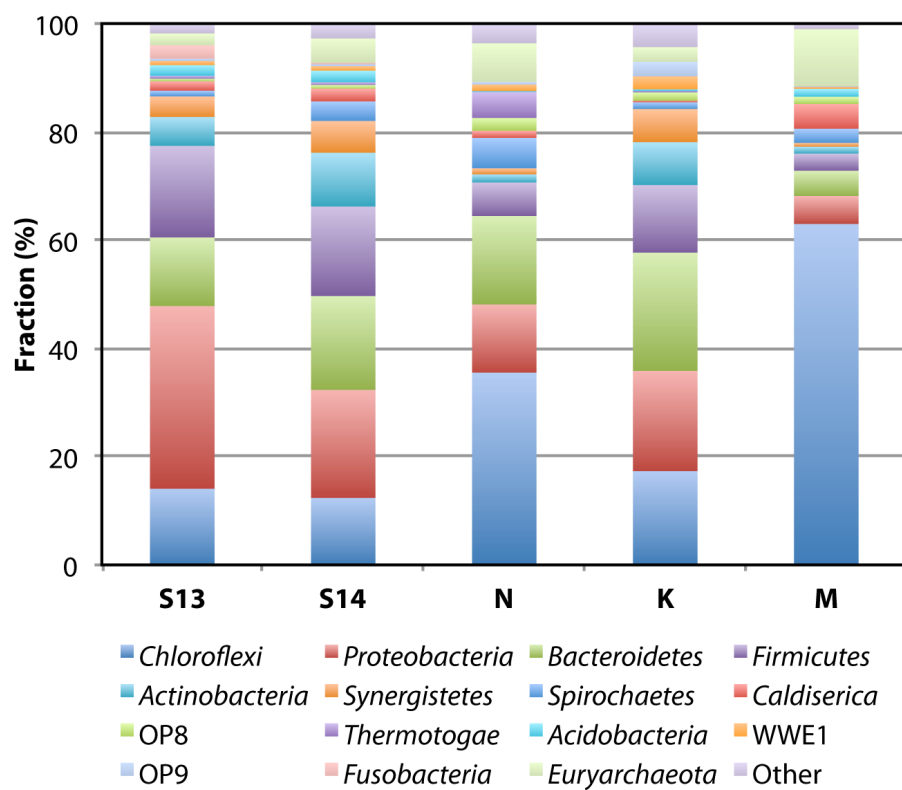

S2 Fig. Prokaryotic community compositions of five sludge samples.

Supplement: S2 Fig — (PDF) [file pone.0172888.s002.pdf]
