# Supplementary material for: Phylogenetic diversity and in situ detection of eukaryotes in anaerobic sludge digesters
Source: PLoS One. 2017 Mar 6;12(3):e0172888. doi: 10.1371/journal.pone.0172888 (PMC5338771; doi:10.1371/journal.pone.0172888)
Supplement: S3 Fig — (PDF) [file pone.0172888.s003.pdf]

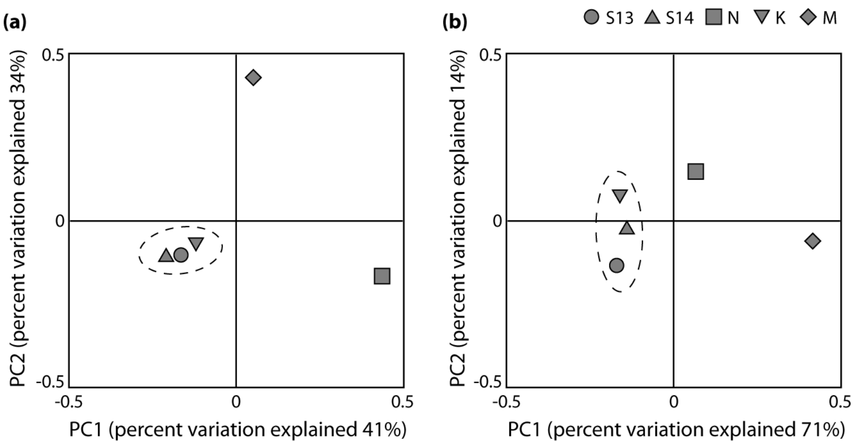

S3 Fig. Principal coordinate analysis (PCoA) of eukaryotic (a) and prokaryotic (b) community structures.
