# Supplementary material for: Phylogenetic diversity and in situ detection of eukaryotes in anaerobic sludge digesters
Source: PLoS One. 2017 Mar 6;12(3):e0172888. doi: 10.1371/journal.pone.0172888 (PMC5338771; doi:10.1371/journal.pone.0172888)
Supplement: S1 Table — (PDF) [file pone.0172888.s004.pdf]

S1 Table. Parameters of full-scale anaerobic digesters.

Sewage works	S		K*1	M	N*2	
	2013	2014			System 1	System 2
Reactor volume (m^3)	3,057		5,200, 3,700 → 5,200	1,800	3,117 → 1,766	5,319 → 3,306
Temperature (˚C)*3	36	37	36-38	32	37.5	37.6
pH*4	7.1	7.2	7.6	6.6	7.1	7.1
HRT (day)	31	29	24*5	48	66*5	78*5
Sewage treatment process	Activated sludge*6		Activated sludge*7	Trickling filter	Activated sludge*7	
Digestion efficiency (%)*4	59	58	60	66	58	65

*1: the digestion system in the K sewage works is sequential. Sludges are subjected either one of two digesters (5,200 m^3 or 3,700 m^3) and effluents are further digested in a second digester (5,200 m^3). The sample was taken from the second digester.

*2: the N sewage works has two sequential digestion systems. The sample from the N sewage works was collected from a pipe, in which effluents from the Systems 1 and 2 are mixed.

*3: monthly average or range in a sampling month.

*4: monthly average.

*5: a total HRT of sequential digesters.

*6: the sewage treatment systems 1-3 employ a pseudo anaerobic-oxic process and the system 4 employs an anaerobic-anoxic-oxic process. The mixture of excess sludge from all systems are subjected to anaerobic digesters.

*7: a conventional activated sludge process.
