# Supplementary material for: Phylogenetic diversity and in situ detection of eukaryotes in anaerobic sludge digesters
Source: PLoS One. 2017 Mar 6;12(3):e0172888. doi: 10.1371/journal.pone.0172888 (PMC5338771; doi:10.1371/journal.pone.0172888)
Supplement: S2 Table — (PDF) [file pone.0172888.s005.pdf]

S2 Table. Primers and probes used in this study.

Name	Sequence (5'-3')	Target	Reference
**For cloning**			
EukA	AAC CTG GTT GAT CCT GCC AGT	Eukaryotic 18S rRNA gene	[1]
EukB	TGA TCC TTC TGC AGG TTC ACC TAC	Eukaryotic 18S rRNA gene	[1]
**For pyrosequencing**			
341f	CCT AYG GGR BGC ASC AG	Prokaryotic 16S rRNA gene	[2]
806r[1) 2)]	GGA CTA CHV GGG THT CTA AT	Prokaryotic 16S rRNA gene	[2]
806r-P[2)]	GGA CTA CCA GGG TAT CTA AG	Prokaryotic 16S rRNA gene	this study
**For quantitative PCR**			
Eub338f ver.1	ACT CCT ACG GGA GGC AGC	Bacterial 16S rRNA gene	[3]
Eub338f ver.2	ACA CCT ACG GGT GGC TGC	Bacterial 16S rRNA gene	[4]
Eub338f ver.3	ACA CCT ACG GGT GGC AGC	Bacterial 16S rRNA gene	[4]
Eub338f ver.4	ACT CCT ACG GGA GGC TGC	Bacterial 16S rRNA gene	[4]
907r	CCG TCA ATT CMT TTR AGT T	Bacterial 16S rRNA gene	[5]
Arc109f	AHD GCT CAG TAA CAC RT	Archaeal 16S rRNA gene	[5]
Arc912r	CCC CCG CCA ATT CCT TTA A	Archaeal 16S rRNA gene	[5]
Euk299f[1)]	GGG TTY GAT TCC GGA GAG	Eukaryotic 18S rRNA gene	[6]
Euk526r[1)]	GGS TGC TGG CAC CAR A	Eukaryotic 18S rRNA gene	[6]
**For FISH**			
Euk516	ACC AGA CTT GCC CTC C	Eukaryotic 18S rRNA gene	[3]
LKM11GP2	GTT CGC ATC TCT ATT TAG CA [3)]	the members in the LKM11 lineage	this study
LKM11GP3	GAG ACT TTC GCC CCT AGT TC [3)]	the members in the LKM11 lineage	this study
A31	CCC TCT CGG GCA AGG ACA AA [3)]	the members in the A31 lineage	this study

1) slightly modified

2) the primers were mixed with the ratio of 30:1 (806r:806r-P)

3) underlined bases are LNA

References

1. Marsh TL, Liu WT, Forney LJ, Cheng H (1998) Beginning a molecular analysis of the eukaryal community in activated sludge. Wat Sci Technol 37(4):455–460.
2. Sundberg C, Al-Soud WA, Larsson M, Alm E, Yekta SS, Svensson BH, et al. (2013) 454 pyrosequencing analyses of bacterial and archaeal richness in 21 full-scale biogas digesters. FEMS Microbiol Ecol 85:612–626.
3. Amann RI, Binder BJ, Olson RJ, Chisholm SW, Devereux R, Stahl DA (1990) Combination of 16S rRNA-targeted oligonucleotide probes with flow cytometry for analyzing mixed microbial populations. Appl Environ Microbiol 56:1919–1925.
4. Daims H, Brühl A, Amann R, Schleifer KH, Wagner M (1999) The domain-specific probe EUB338 is insufficient for the detection of all *Bacteria*: Development and evaluation of a more comprehensive probe set. Syst Appl Microbiol 22:434–444.
5. Miyashita A, Mochimaru H, Kazama H, Ohashi A, Yamaguchi T, Nunoura T, et al. (2009) Development of 16S rRNA gene-targeted primers for detection of archaeal anaerobic methanotrophs (ANMEs). FEMS Microbiol Lett 297:31–37.
6. Moreno AM, Matz C, Kjelleberg S, Manefield M (2010) Identification of ciliate grazers of autotrophic bacteria in ammonia-oxidizing activated sludge by RNA stable isotope probing. Appl Environ Microbiol 76:2203–2211.
