# Supplementary material for: Phylogenetic diversity and in situ detection of eukaryotes in anaerobic sludge digesters
Source: PLoS One. 2017 Mar 6;12(3):e0172888. doi: 10.1371/journal.pone.0172888 (PMC5338771; doi:10.1371/journal.pone.0172888)
Supplement: S3 Table — (PDF) [file pone.0172888.s006.pdf]

# S3 Table. List of operational taxonomic units (OTUs)

| Out No. | No.of clone | Kngdom / Superphylum | Phylum | | acc. number of close relative | Sequence identity(%) | Accession No. |
|---|---|---|---|---|---|---|---|
| Sample name : S13 | | | | | | | |
| 1 | 11 | Alveolata | Perkinsozoa | A31 | | | LC109053 |
| 2 | 7 | Fungi | Cryptomycota | LKM11 | | | LC109054 |
| 3 | 4 | Fungi | Cryptomycota | LKM11 | | | LC109055 |
| 4 | 3 | Fungi | Cryptomycota | LKM11 | | | LC109056 |
| 5 | 2 | Fungi | Zoopagales | – | | | LC109057 |
| 6 | 2 | Fungi | Cryptomycota | LKM11 | | | LC109058 |
| 7 | 2 | Metazoa | Platyhelminthes | *Gieysztoria sp.* | KC529464 | 99 | LC109059 |
| 8 | 1 | Archaeplastida | Chlorophyta | AN1-3 | | | LC109060 |
| 9 | 1 | Alveolata | Ciliophora | *Acaryophrya sp.* | KF733758 | 99 | LC109061 |
| 10 | 1 | Fungi | Ascomycota | family Dipodascaceae | | | LC109062 |
| 11 | 1 | Fungi | Ascomycota | *Penicillium chrysogenum* | AF411201 | 99 | LC109063 |
| 12 | 1 | Fungi | Ascomycota | *Exophiala equina* | JN856017 | 99 | LC109064 |
| 13 | 1 | Fungi | Cryptomycota | LKM11 | | | LC109065 |
| 14 | 1 | Fungi | Ascomycota | family Dipodascaceae | | | LC109066 |
| 15 | 1 | Fungi | Cryptomycota | LKM11 | | | LC109067 |
| 16 | 1 | Fungi | Ascomycota | family Dipodascaceae | | | LC109068 |
| 17 | 1 | Fungi | Basidomycota | *Trichosporon cutaneum* | X60182 | 99 | LC109069 |
| 18 | 1 | Fungi | Ascomycota | *Candida sp.* | HM161746 | 99 | LC109070 |
| 19 | 1 | Archaeplastida | Chlorophyta | family Chlorellaceae | | | LC109071 |
| 20 | 1 | Metazoa | Arthropoda | *Rhizoglyphus sp.* | HM070357 | 98 | LC109072 |
| 21 | 1 | Archaeplastida | Chlorophyta | family Chlorellaceae | | | LC109073 |
| 22 | 1 | Archaeplastida | Chlorophyta | family Chlorellaceae | | | LC109074 |
| 23 | 1 | Fungi | Ascomycota | family Dipodascaceae | | | LC109075 |
| 24 | 1 | Fungi | Ascomycota | family Dipodascaceae | | | LC109076 |
| 25 | 1 | Fungi | Ascomycota | family Dipodascaceae | | | LC109077 |
| 26 | 1 | Metazoa | Gastrotricha | *Chaetonotus cf.* | JQ798603 | 99 | LC109078 |
| 27 | 1 | Archaeplastida | Chlorophyta | family Chlorellaceae | | | LC109079 |
| 28 | 1 | Stramenopiles | Hyphochytriomycetes | *Rhizidiomyces apophysatus* | AF163295 | 97 | LC109080 |
| Sample name : S14 | | | | | | | |
| 1 | 9 | Alveolata | Perkinsozoa | A31 | | | LC109081 |
| 2 | 7 | Fungi | Cryptomycota | LKM11 | | | LC109082 |
| 3 | 5 | Fungi | Cryptomycota | LKM11 | | | LC109083 |
| 4 | 3 | Fungi | Ascomycota | family Dipodascaceae | | | LC109084 |
| 5 | 2 | Fungi | Ascomycota | family Dipodascaceae | | | LC109085 |
| 6 | 2 | Fungi | Ascomycota | family Dipodascaceae | | | LC109086 |
| 7 | 2 | Fungi | Chytridomycota | *Hyaloraphidium curvatum* | NG_017172 | 97 | LC109087 |
| 8 | 2 | Fungi | Cryptomycota | LKM11 | | | LC109088 |
| 9 | 2 | Metazoa | Rotifera | *Brachionus calyciflorus* | KF141790 | 99 | LC109089 |
| 10 | 2 | Fungi | Ascomycota | family Dipodascaceae | | | LC109090 |
| 11 | 1 | Fungi | Cryptomycota | LKM11 | | | LC109091 |
| 12 | 1 | Fungi | Cryptomycota | LKM11 | | | LC109092 |
| 13 | 1 | Archaeplastida | Chlorophyta | *Prototheca zopfii* | X63519 | 99 | LC109093 |
| 14 | 1 | Fungi | Cryptomycota | LKM11 | | | LC109094 |
| 15 | 1 | Fungi | Cryptomycota | LKM11 | | | LC109095 |
| 16 | 1 | Alveolata | Ciliophora | subclass Peritrichia | | | LC109096 |
| 17 | 1 | Alveolata | Ciliophora | *Vorticellides aquadulcis* | JQ723990 | 99 | LC109097 |
| 18 | 1 | Fungi | Cryptomycota | LKM11 | | | LC109098 |
| 19 | 1 | Archaeplastida | Chlorophyta | family Chlorellaceae | | | LC109099 |
| 20 | 1 | Fungi | Cryptomycota | LKM11 | | | LC109100 |
| 21 | 1 | Rhizaria | Cercozoa | *Rhogostoma minus* | LC032468 | 98 | LC109101 |
| 22 | 1 | Fungi | Ascomycota | class saccharomycetes | | | LC109102 |

## S3 Table. The list of operational taxonomic units (OTUs) *(continued)*

| Out No. | No.of clone | Kngdom / Superphylum | Phylum | | acc. number of close relative | Sequence identity(%) | Accession No. |
|---|---|---|---|---|---|---|---|
| **Sample name : N** | | | | | | | |
| 1 | 20 | Fungi | Cryptomycota | LKM11 | | | LC109037 |
| 2 | 10 | Fungi | Cryptomycota | LKM11 | | | LC109038 |
| 3 | 9 | Fungi | Cryptomycota | LKM11 | | | LC109039 |
| 4 | 3 | Fungi | Cryptomycota | LKM11 | | | LC109040 |
| 5 | 2 | Fungi | Cryptomycota | LKM11 | | | LC109041 |
| 6 | 1 | Fungi | Cryptomycota | LKM11 | | | LC109042 |
| 7 | 1 | Fungi | Cryptomycota | LKM11 | | | LC109043 |
| 8 | 1 | Fungi | Ascomycota | *Phoma sp.* | EF532930 | 99 | LC109044 |
| 9 | 1 | Fungi | Cryptomycota | LKM11 | | | LC109045 |
| 10 | 1 | Viridiplantae | Chlorophyta | *Dictyochloropsis symbiontica* | GU017651 | 99 | LC109046 |
| 11 | 1 | Animalia | Gastrotricha | *Chaetonotus cf.* | JQ798603 | 99 | LC109047 |
| 12 | 1 | Fungi | Ascomycota | family Dipodascaceae | | | LC109048 |
| 13 | 1 | Fungi | Cryptomycota | LKM11 | | | LC109049 |
| 14 | 1 | Fungi | Basidomycota | *Lentinus sp.* | GQ899200 | 99 | LC109050 |
| 15 | 1 | Fungi | Ascomycota | family Dipodascaceae | | | LC109051 |
| 16 | 1 | Fungi | Cryptomycota | LKM15 | | | LC109052 |
| **Sample name : K** | | | | | | | |
| 1 | 16 | Fungi | Cryptomycota | LKM11 | | | LC109023 |
| 2 | 10 | Alveolata | Perkinsozoa | A31 | | | LC109024 |
| 3 | 8 | Fungi | Cryptomycota | LKM11 | | | LC109025 |
| 4 | 4 | Fungi | Cryptomycota | LKM11 | | | LC109026 |
| 5 | 3 | Viridiplantae | Chlorophyta | family Chlorellaceae | | | LC109027 |
| 6 | 2 | Viridiplantae | Chlorophyta | AN1-3 | | | LC109028 |
| 7 | 1 | Fungi | Cryptomycota | LKM11 | | | LC109029 |
| 8 | 1 | Fungi | Cryptomycota | LKM11 | | | LC109030 |
| 9 | 1 | Metazoa | Arthropoda | *Boletoglyphus extremiorientali* | JQ000111 | 98 | LC109031 |
| 10 | 1 | Fungi | Ascomycota | *Exophiala equina* | JN856017 | 99 | LC109032 |
| 11 | 1 | Alveolata | Perkinsozoa | A31 | | | LC109033 |
| 12 | 1 | Fungi | Cryptomycota | LKM11 | | | LC109034 |
| 13 | 1 | Fungi | Cryptomycota | LKM11 | | | LC109035 |
| 14 | 1 | Viridiplantae | Chlorophyta | family Chlorellaceae | | | LC109036 |
| **Sample name : M** | | | | | | | |
| 1 | 6 | Fungi | Cryptomycota | LKM11 | | | LC108996 |
| 2 | 6 | Metazoa | Arthropoda | *Allonothrus russeolus* | AF022025 | 99 | LC108997 |
| 3 | 3 | Viridiplantae | Chlorophyta | AN1-3 | | | LC108998 |
| 4 | 2 | Amoebozoa | Discosea | order Centramoebida | | | LC108999 |
| 5 | 2 | Viridiplantae | Chlorophyta | family Chlorellaceae | | | LC109000 |
| 6 | 2 | Viridiplantae | Chlorophyta | family Chlorellaceae | | | LC109001 |
| 7 | 2 | Fungi | Ascomycota | superphylum Pezizomycotina | | | LC109002 |
| 8 | 2 | Alveolata | Apicomplexa | order Eugregarinorida | | | LC109003 |
| 9 | 2 | Fungi | Basidomycota | *Glaciozyma antarctica* | DQ525623 | 99 | LC109004 |
| 10 | 1 | Viridiplantae | Chlorophyta | AN1-3 | | | LC109005 |
| 11 | 1 | Viridiplantae | Chlorophyta | family Chlorellaceae | | | LC109006 |
| 12 | 1 | Viridiplantae | Chlorophyta | *Prototheca zopfii* | X63519 | 99 | LC109007 |
| 13 | 1 | Fungi | Ascomycota | *Candida tropicalis* | M55527 | 99 | LC109008 |
| 14 | 1 | Fungi | Basidomycota | *Trichosporon scarabaeorum* | JN939434 | 99 | LC109009 |
| 15 | 1 | Fungi | Ascomycota | *Xenobotrytis acaducospora* | EU541483 | 99 | LC109010 |
| 16 | 1 | Fungi | Cryptomycota | LKM11 | | | LC109011 |
| 17 | 1 | Fungi | Cryptomycota | LKM11 | | | LC109012 |
| 18 | 1 | Fungi | Cryptomycota | LKM11 | | | LC109013 |
| 19 | 1 | Metazoa | Arthropoda | *Naiadacarus arboricola* | JQ000114 | 98 | LC109014 |
| 20 | 1 | Metazoa | Tardigrada | *Hypsibius convergens* | FJ435726 | 98 | LC109015 |
| 21 | 1 | Fungi | Ascomycota | family Dipodascaceae | | | LC109016 |
| 22 | 1 | Amoebozoa | Discosea | order Dactylopodida | | | LC109017 |
| 23 | 1 | Rhizaria | Cercozoa | class Phytomyxea | | | LC109018 |
| 24 | 1 | Viridiplantae | Chlorophyta | *Scenedesmus armatus* | KF864474 | 99 | LC109019 |
| 25 | 1 | Viridiplantae | Chlorophyta | *Coelastrella saipanensis* | AB055800 | 99 | LC109020 |
| 26 | 1 | Viridiplantae | Chlorophyta | family Chlorellaceae | | | LC109021 |
| 27 | 1 | Fungi | Ascomycota | family Dipodascaceae | | | LC109022 |
