# Supplementary material for: Phylogenetic diversity and in situ detection of eukaryotes in anaerobic sludge digesters
Source: PLoS One. 2017 Mar 6;12(3):e0172888. doi: 10.1371/journal.pone.0172888 (PMC5338771; doi:10.1371/journal.pone.0172888)
Supplement: S4 Table — (PDF) [file pone.0172888.s007.pdf]

S4 Table. The numbers of sequence reads and diversity indices of prokaryotic community.

Sample name	S13	S14	N	K	M
No. of seq. reads	11,226	4,484	10,617	9,166	9,867
No. of OTUs	753	505	583	546	334
Chao1	1,178	823	842	834	522
Shannon	7.46	7.13	6.25	6.63	4.52
Coverage	0.98	0.95	0.98	0.98	0.99
